# Supplementary material for: Fetal Myocardial Expression of GLUT1: Roles of BPA Exposure and Cord Blood Exosomes in a Rat Model
Source: Cells. 2022 Oct 11;11(20):3195. doi: 10.3390/cells11203195 (PMC9601122; doi:10.3390/cells11203195)
Supplement: Supplementary file 1 [file cells-11-03195-s001.zip › cells-1861219-supplementary.pdf]

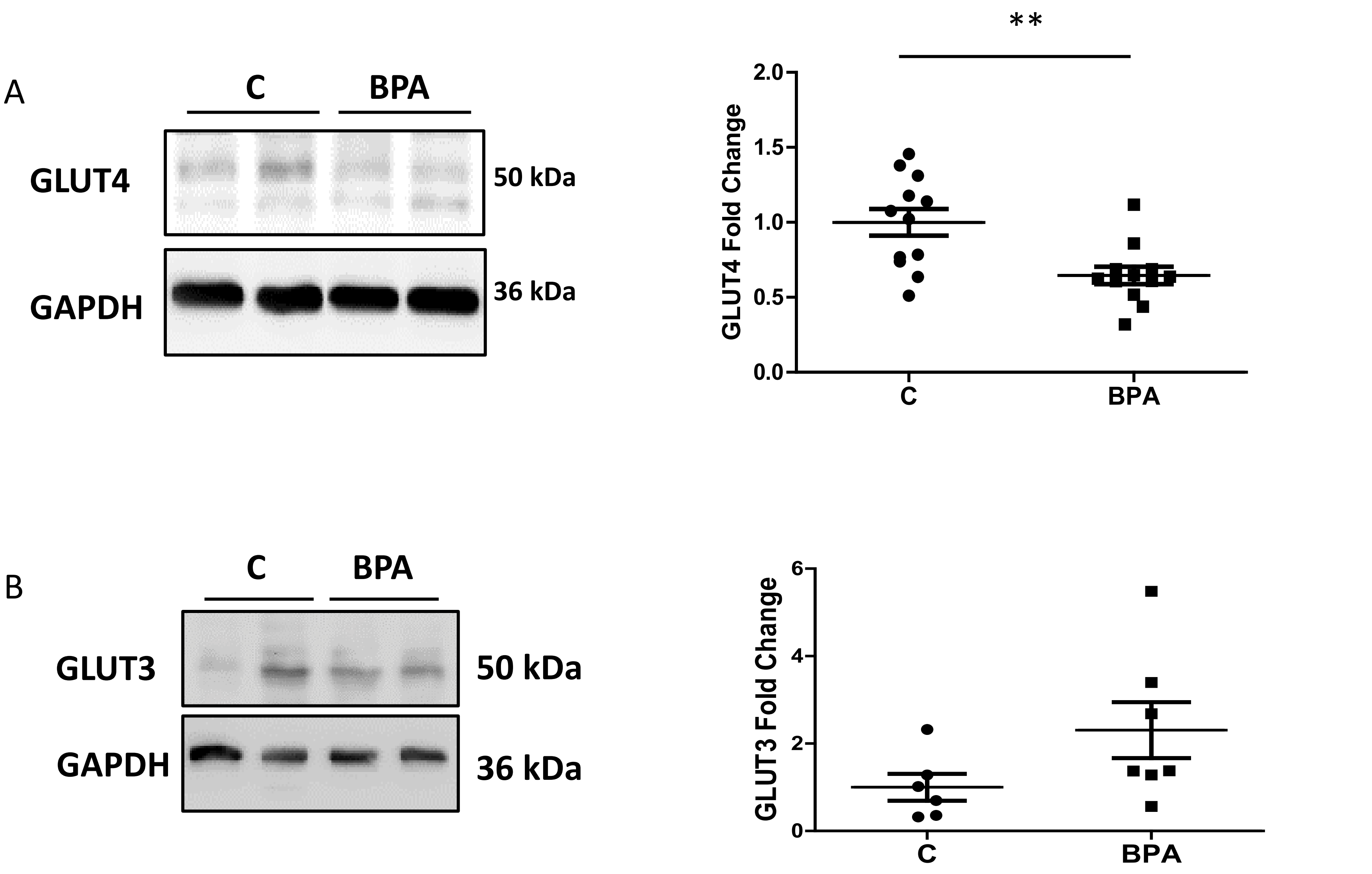

Figure S1. Representative WB and corresponding densitometry for GLUT4 (A) and GLUT3 (B) in lysates of fetal hearts obtained from BPA feed and control rats.

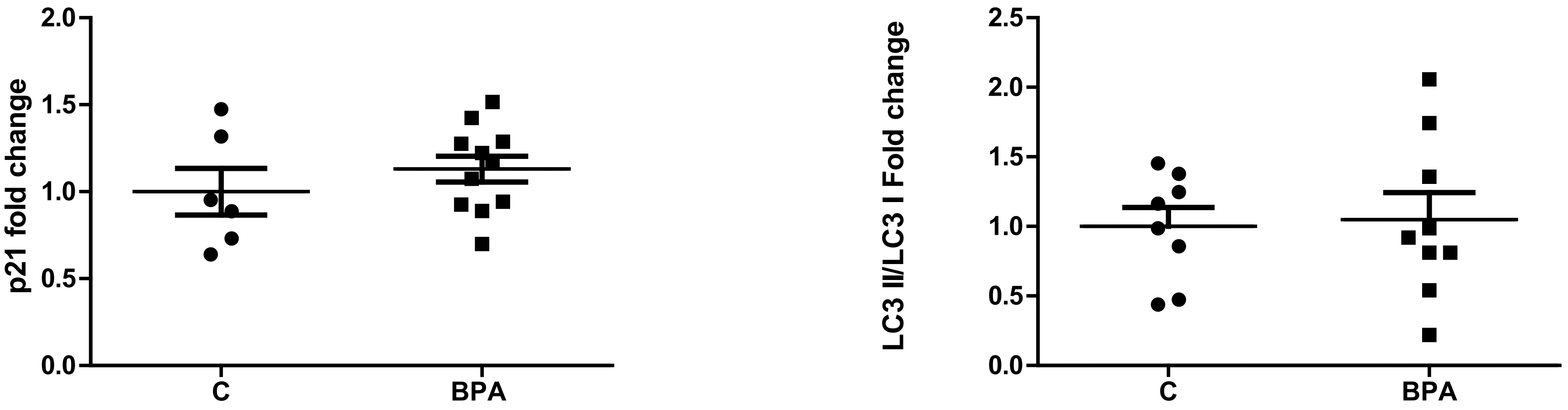

Figure S2. Densitometry for p21 and LC3 in lysates of fetal hearts obtained from BPA feed and control rats.

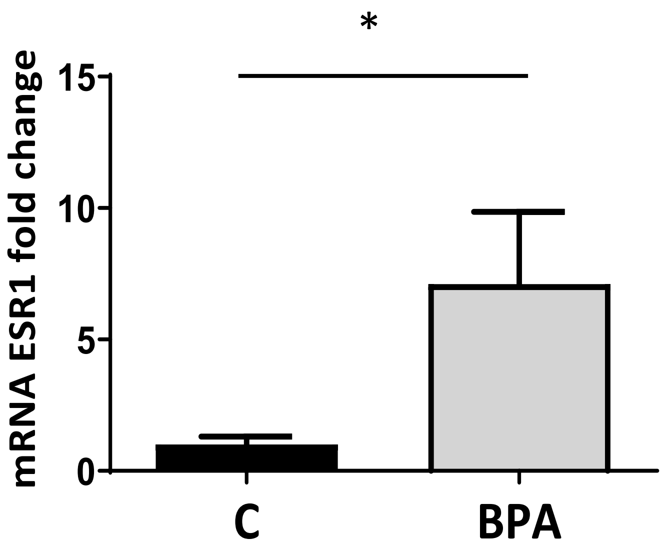

Figure S3. Estrogen receptor alpha (ESR1) mRNA expression in fetal hearts obtained from BPA feed and control rats.

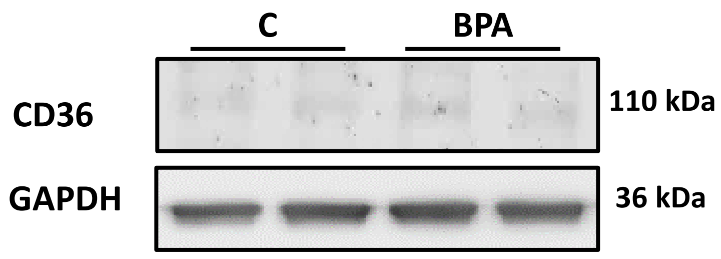

Figure S4. Representative WB for CD36 in lysates of fetal heart organotypic culture treated with BPA or control.

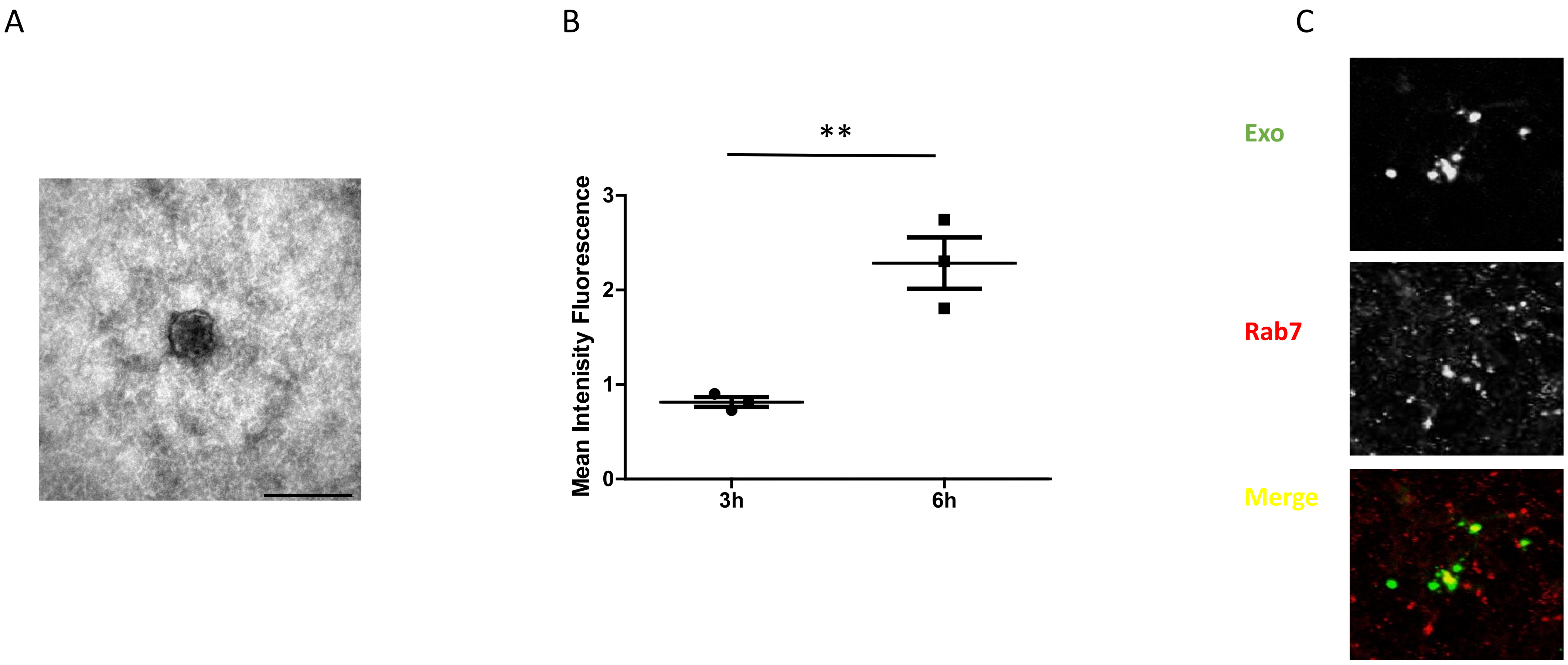

Figure S5. TEM analysis (A) and uptake (B and C) of cord blood exosomes.
